# 1734. Susceptibility of Cefiderocol between US Census Regions against Gram-Negative Organisms collected from the SENTRY Surveillance Program: 2020-2021

**DOI:** 10.1093/ofid/ofac492.1364

**Published:** 2022-12-15

**Authors:** Frank H Kung, Sean Nguyen, Christine M Slover, Dee Shortridge, Jennifer M Streit, Roger Echols, Roger Echols, Roger Echols, Miki Takemura, Yoshinori Yamano

**Affiliations:** Shionogi Inc, Florham Park, New Jersey; Shionogi Inc, Florham Park, New Jersey; Shionogi Inc., Florham Park, New Jersey; JMI Laboratories, North Liberty, Iowa; JMI Laboratories, North Liberty, Iowa; Infectious Disease Drug Development Consulting, Easton, Connecticut; Infectious Disease Drug Development Consulting, Easton, Connecticut; Infectious Disease Drug Development Consulting, Easton, Connecticut; Shionogi & Co., Ltd, Toyonaka, Osaka, Japan; Shionogi & Co., Ltd., Toyonaka, Osaka, Japan

## Abstract

**Background:**

Cefiderocol (CFDC) is a siderophore-conjugated cephalosporin with broad activity against Gram-negative (GN) bacteria, including multidrug-resistant organisms. GN bacteria such as Enterobacterales (ENT)*, Pseudomonas aeruginosa* (PsA)*, Acinetobacter baumannii* complex (ABC), and *Stenotrophomonas maltophilia* (StM) can be challenging to treat and are often carbapenem-resistant (CR). Regional susceptibility of CFDC and comparators were investigated against US GN isolates collected in 2020-2021 as part of the SENTRY Antimicrobial Surveillance Program.

**Methods:**

GN pathogens were consecutively collected from 32 US hospitals between 2020 to 2021. Susceptibility testing was performed using the broth microdilution method. CFDC was tested in iron-depleted cation-adjusted Mueller-Hinton broth. FDA or CLSI breakpoints were used where available. Other agents tested included the beta-lactam/beta-lactamase inhibitor (BL/BLI) combinations ceftazidime-avibactam, ceftolozane-tazobactam, imipenem-relebactam, meropenem-vaborbactam, piperacillin-tazobactam, and ampicillin-sulbactam. CR-PsA, or CRAB was defined as meropenem resistant while CRE was defined as imipenem or meropenem resistant by CLSI breakpoints.

**Results:**

A total of 8328 ENT, 2241 PsA, 586 ABC, and 404 StM were collected. For ENT and PsA, CFDC susceptibility between the 9 US Census regions remained above 98% susceptible by CLSI or FDA breakpoints in all regions (**Table 1**). Amongst the BL/BLI combinations, the majority had >90% susceptibility across regions except for piperacillin-tazobactam with 79.7% for ENT and 65.9% for PsA in the MidAtlantic by FDA breakpoints. For ABC and StM, CFDC susceptibility was > 88% across all regions. For CR pathogens, 96 CRE, 327 CR-PsA, and 199 CRAB were collected. CFDC had >87.5% susceptibility in all regions for CRE and CR-PSA. For CRAB, 4 regions were > 87.5% by FDA breakpoints and the lowest was 63.6% in 11 isolates in the Mountain region **(Table 2)**. For CRAB, every BL/BLI had susceptibility ≤27.3% in every region. Ampicillin-sulbactam had 27.3% susceptibility in the Mountain region.

**Table 1:**
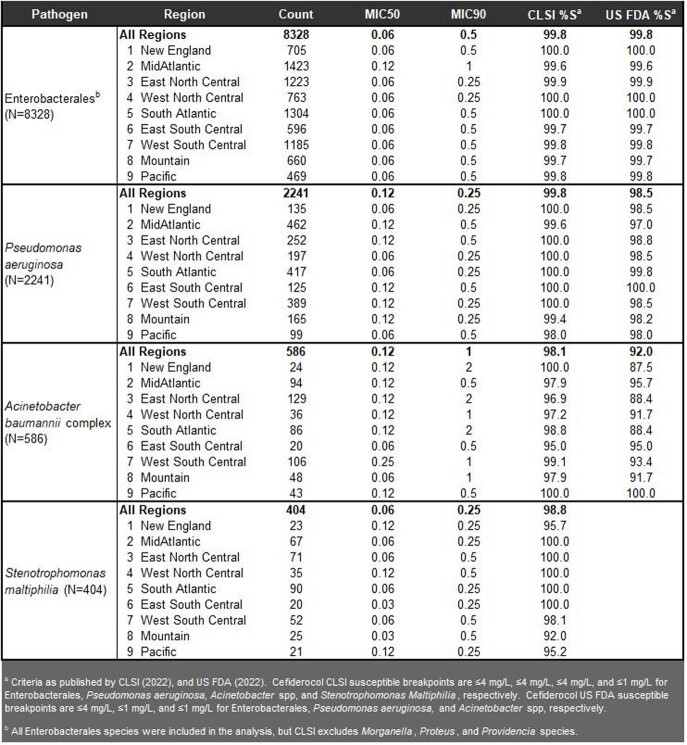

Susceptibility of Cefiderocol against Enterobacterales, Pseudomonas aeruginosa, Acinetobacter baumannii complex and Stenotrophomonas maltiphilia isolates in the cefiderocol program collected from medical centers in the USA

**Table 2:**
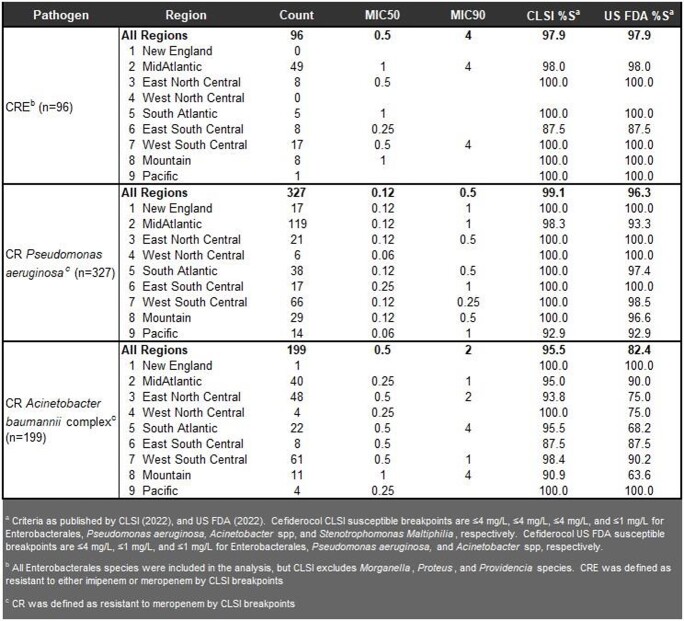

Susceptibility of cefiderocol against CRE, CR Pseudomonas aeruginosa, and CR Acinetobacter baumannii complex isolates in the cefiderocol program collected from medical centers in the USA

**Conclusion:**

US GN isolates, including CR pathogens, had high susceptibilities to CFDC across the US census regions. CFDC remains an important treatment option for GN infections in all US census regions.

**Disclosures:**

**Frank H. Kung, PhD**, Shionogi Inc: Employee **Sean Nguyen, n/a**, Shionogi: Employee **Christine M. Slover, PharmD**, Shionogi: Employee **Dee Shortridge, PhD**, AbbVie: Grant/Research Support|JMI Laboratory: Employee|Melinta: Grant/Research Support|Menarini: Grant/Research Support|Shionogi: Grant/Research Support **Jennifer M. Streit, BS, MT(ASCP)**, Cidara: Grant/Research Support|GSK: Grant/Research Support|Melinta: Grant/Research Support|Shionogi: Grant/Research Support **Roger Echols, MD**, Shionogi: Advisor/Consultant **Roger Echols, MD**, Shionogi: Advisor/Consultant **Roger Echols, MD**, Shionogi: Advisor/Consultant **Miki Takemura, n/a**, Shionogi: Employee **Yoshinori Yamano, PhD**, Shionogi: Employee.

